# Program synergies and social relations: implications of integrating HIV testing and counselling into maternal health care on care seeking

**DOI:** 10.1186/s12889-014-1336-3

**Published:** 2015-01-21

**Authors:** Selena J An, Asha S George, Amnesty LeFevre, Rose Mpembeni, Idda Mosha, Diwakar Mohan, Ann Yang, Joy Chebet, Chrisostom Lipingu, Japhet Killewo, Peter Winch, Abdullah H Baqui, Charles Kilewo

**Affiliations:** International Center for Maternal and Newborn Health, Department of International Health, Johns Hopkins Bloomberg School of Public Health, 615 N Wolfe Street, Baltimore, MD 21205 USA; Department of Epidemiology and Biostatistics, Muhimbili University of Health and Allied Sciences, PO Box 65015, Dar es Salaam, Tanzania; Department of Behavioural Sciences, Muhimbili University of Health and Allied Sciences, PO Box 65015, Dar es Salaam, Tanzania; Department of Obstetrics and Gynecology, Muhimbili University of Health and Allied Sciences, PO Box 65015, Dar es Salaam, Tanzania; Jhpiego, Dar es Salaam, Tanzania

**Keywords:** ANC, HIV testing and counselling, Integration, Care seeking, Patient-provider interaction, Stigma

## Abstract

**Background:**

Women and children in sub-Saharan Africa bear a disproportionate burden of HIV/AIDS. Integration of HIV with maternal and child services aims to reduce the impact of HIV/AIDS. To assess the potential gains and risks of such integration, this paper considers pregnant women’s and providers’ perceptions about the effects of integrated HIV testing and counselling on care seeking by pregnant women during antenatal care in Tanzania.

**Methods:**

From a larger evaluation of an integrated maternal and newborn health care program in Morogoro, Tanzania, this analysis included a subset of information from 203 observations of antenatal care and interviews with 57 providers and 190 pregnant women from 18 public health centers in rural and peri-urban settings. Qualitative data were analyzed manually and with Atlas.ti using a framework approach, and quantitative data of respondents’ demographic information were analyzed with Stata 12.0.

**Results:**

Perceptions of integrating HIV testing with routine antenatal care from women and health providers were generally positive. Respondents felt that integration increased coverage of HIV testing, particularly among difficult-to-reach populations, and improved convenience, efficiency, and confidentiality for women while reducing stigma. Pregnant women believed that early detection of HIV protected their own health and that of their children. Despite these positive views, challenges remained. Providers and women perceived opt out HIV testing and counselling during antenatal services to be compulsory. A sense of powerlessness and anxiety pervaded some women’s responses, reflecting the unequal relations, lack of supportive communications and breaches in confidentiality between women and providers. Lastly, stigma surrounding HIV was reported to lead some women to discontinue services or seek care through other access points in the health system.

**Conclusion:**

While providers and pregnant women view program synergies from integrating HIV services into antenatal care positively, lack of supportive provider-patient relationships, lack of trust resulting from harsh treatment or breaches in confidentiality, and stigma still inhibit women’s care seeking. As countries continue rollout of Option B+, social relations between patients and providers must be understood and addressed to ensure that integrated delivery of HIV counselling and services encourages women’s care seeking in order to improve maternal and child health.

## Background

In sub-Saharan Africa, women comprised 60% of people living with human immunodeficiency virus and acquired immune deficiency syndrome (HIV/AIDS) in 2011, and AIDS was the leading cause of death among mothers [1]. The disproportionate burden of HIV in women has implications for not only their health but also the health of their children. Ninety one percent of children under 15 years living with HIV in 2011 were in sub-Saharan Africa [1]. In 2012, the prevalence of HIV/AIDS in Tanzania was 5.1% among adults generally, 6.2% among women of reproductive age, and 3.2% among pregnant women [2]. An estimated 70,000 to 80,000 newborns were at risk of acquiring HIV every year during pregnancy or delivery, or via breastfeeding [3]. One programmatic response to the vulnerability of women and children to HIV has been the package of interventions focused on the prevention of mother-to-child transmission (PMTCT) [4]. PMTCT is envisioned as a cascade of services throughout the reproductive, maternal, newborn, and child health spectrum that entails a series of services, including counselling, testing and treatment, delivered at multiple time points throughout a woman’s interaction with the health system.

In the last decade, integration of PMTCT services into routine maternal and child health (MCH) services has been a key strategy in responding to HIV/AIDS in low-resource settings [4,5]. By strengthening the linkages between MCH and PMTCT, integration is believed to improve the coverage of HIV testing and treatment, leading to earlier treatment for those who need it and an opportunity for HIV-positive pregnant mothers to receive prophylaxis so that transmission is prevented. In addition, integration is also believed to strengthen basic health systems services, improve the efficiency of service delivery, and increase the effectiveness of health interventions [4–7].

In Tanzania, HIV integration entailed provision of HIV testing, counselling, and treatment for HIV-positive pregnant women during antenatal care (ANC). HIV testing and counselling were integrated into MCH services starting in 2007 on an “opt out” basis (Table 1) [8]. Under these guidelines, prophylaxis for prevention of vertical transmission was part of reproductive and child health clinics (RCH), while care and treatment for HIV-positive pregnant women remained in care and treatment centers (CTC) [8]. In October 2013, Tanzania began the implementation of Option B+ as recommended by the World Health Organization (WHO) [9]. Under Option B+, pregnant and lactating mothers who test positive for HIV are eligible for antiretroviral therapy (ART) in RCH wards, regardless of their CD4 count and stage.

**Table 1:** **PMTCT policies in Tanzania**

**Year**	**Source**	**Policy content**
2000-2002	Pilot PMTCT Program	• Short course regimen for preventing mother-to-child transmission in four referral hospitals and one regional hospital
		• Use of AZT short course from 36 weeks to delivery
2004	First national PMTCT guidelines for scale up	• Scale up from 5 pilot testing sites to the whole country (1347 sites across the country by 2006)
		• sdNVP during labor and delivery
2007	Second national PMTCT guidelines for scale up	• Provider initiated testing and counselling in antenatal visits in an “opt out” system
		• PMTCT remained in parallel to Care and Treatment Centers (CTC), where eligible mothers received care
		• Change of regimen from sdNVP to AZT from 28 weeks of pregnancy until labor and delivery for PMTCT
2011	Third national PMTCT guidelines for scale up	• Tanzania adopts option A of 2010 WHO guidelines (use of ARV drugs for treating pregnant women and preventing mother-to-child transmission of HIV)
		• Engagement with, testing of, and counselling partners at health facilities
		• PMTCT program expanded to 3420 sites in the country
2013	Fourth national PMTCT guidelines Option B/B+	• All HIV-infected pregnant and lactating mothers, regardless of CD4 count, eligible for lifelong treatment with antiretroviral drugs
		• Care and treatment integrated into RCH wards

Connecting HIV-positive mothers who tested during antenatal care to ART and following up with them after delivery has been challenging [10]. While some studies suggested that higher utilization of MCH services could lead to increased utilization of integrated HIV services [11,12], evidence for the uptake and adherence to ART among pregnant women and infants remained mixed [13–15]. At the same time, literature on client satisfaction with integrated HIV testing and counselling programs showed that most clients were satisfied with the services provided under integration, including counselling, wait time, and providers [16,17]. From a provider perspective, a study in rural Kenya found that health workers viewed integration as a mostly positive development, as this approach enhanced service provision, improved patient-provider relationships, and increased likelihood of HIV-positive women’s enrolment into HIV care by decreasing stigma [18].

Given the resources devoted to integration of HIV services in the maternal, newborn and child health (MNCH) spectrum of care and to inform future policies in this area, we aim in this paper to understand providers’ and pregnant women’s perceptions of the integration of HIV testing and counselling within routine antenatal care and the effects of integration on care seeking. We report the characteristics of respondents and antenatal care health workers to provide some contextual background for the findings. We then detail the views of pregnant women and providers on the generally positive program synergies from integrating HIV testing and counselling into antenatal care and then discuss the remaining challenges and concerns reflecting the social relations that underpin service delivery.

## Methods

### Study site

Populated with 44.8 million people and located in east Africa, Tanzania is a low-income country with a per capita gross national income of 540 U.S. dollars [19]. With regards to maternal health, focused antenatal care (FANC) guidelines in 2002 reduced the frequency of facility visits from monthly to a minimum of four times with new counselling and clinical services (Table 2) [20,21]. Between 2005 and 2010, 95.8% of pregnant women in mainland Tanzania made at least one antenatal care visit with skilled providers. Yet, only 42.7% of women in mainland Tanzania made four or more antenatal care visits, and half of them made their first visit during the fifth month of pregnancy [22]. While women can and do access antenatal care in facilities, challenges in terms of continuity and quality remain. Overall, 23.1% of women reported having at least one problem in accessing health care [22].

**Table 2:** **Integrated HIV and ANC services in Tanzania**

**Focused antenatal care checklist**				
**Parameter**	**First visit <16 weeks**	**Second visit 20–24 weeks**	**Third visit 28–32 weeks**	**Fourth visit 36 weeks**
**Laboratory investigations, blood**				
Haemoglobin	✓	✓	✓	✓
Grouping and rhesus factor	✓			
RPR	✓			
HIV testing	✓			
**Client education and counselling (for the couple)**				
Process of pregnancy and complications	✓	✓	✓	✓
Diet and nutrition	✓	✓	✓	✓
Rest and exercise in pregnancy	✓	✓	✓	✓
Personal hygiene	✓			
Danger signs in pregnancy	✓	✓	✓	✓
Use of drugs in pregnancy	✓	✓	✓	✓
Effects of STI/HIV/AIDS	✓	✓	✓	✓
Voluntary counselling and testing for HIV	✓			
Care of breasts and breast feeding	✓			✓
Symptoms/signs of labour			✓	✓
Plans of delivery (emergency preparedness, place of delivery, transportation, financial arrangements)	✓	✓	✓	✓
Plans for postpartum care			✓	✓
Family planning			✓	✓
Harmful habits (e.g. smoking, drug abuse, alcoholism)	✓	✓	✓	✓
Schedule of return visit	✓	✓	✓	✓

Source: Adapted from von Both C, Fleba S, Makuwani A, Mpembeni R, Jahn A. How much time do health services spend on antenatal care? Implications for the introduction of the focused antenatal care model in Tanzania. BMC Pregnancy and Childbirth 2006, 6(22).

Morogoro is one of 30 regions in Tanzania, located about 200 kilometers southwest of Dar es Salaam [23]. With a population of 2.2 million and a population density of 31 inhabitants per square kilometer, Morogoro region is among Tanzania’s largest and least densely populated regions. According to a 2002 census, more people live in rural areas (73%) than in urban areas (27%) in Morogoro, similar to most regions in Tanzania [24]. Regional averages for education, poverty and care seeking are also similar to national averages. With regards to HIV, 67.1% of women of reproductive age and 49.8% of men between 15–49 in Morogoro have ever tested for HIV, while 5.3% of women of reproductive age and 2.1% of men between the ages of 15–49 are HIV-positive [2].

### Study design

As part of a three-year evaluation of a maternal and newborn health care program implemented by the Ministry of Health and Social Welfare (MoHSW) and MAISHA through Jhpiego in Morogoro, Tanzania, all 18 government health centers in four rural and peri-urban districts (Gairo, Kilosa,^a^ Morogoro District Council, Mvomero, and Ulanga) were chosen for a cross-sectional health facility assessment.

### Data collection

A team of six research assistants received training over six days that included research ethics and techniques, project objectives, overview of instruments, and two days of pilot testing in health care facilities. Data collection proceeded from September to early December 2012. In each health facility, data were collected over a period of two days.

Prior to the start of data collection, study personnel visited each health facility in-charge to brief him or her on data collection objectives and coordinate data collection on the days when antenatal and postnatal services were provided (Table 3). At each health facility, the first ten pregnant women attending routine antenatal services were approached for their participation and consent to the study and then subsequently observed and interviewed.

**Table 3:** **Data sources included in MNCH facility survey**

**Data source**	**Sampling**	**Final sample**
Facility observation checklists and interviews with facility in charge	Census of health centers	18
ANC provider interviews quantitative	Sub-analysis of 88 RCH providers interviewed based on availability on day of visit and provision of antenatal care in the preceding 7 days	65
ANC provider interviews qualitative	Sub-sample of 88 RCH providers interviewed based on availability on day of visit, receipt of Jhpiego PNC training, and years of service as a provider in the facility; average of 3 per facility	57
ANC sessions observed	Quota based on availability on day of visit, average of 10 per facility, total approved target sample of 240	203; 8 refusals
ANC women exit interviews	Sub-sample of those who consented to observation of ANC sessions	196; 7 refusals

At least five providers per facility providing antenatal and postnatal services during the day shift were administered a structured quantitative survey. A sub-sample of about three providers per facility were then chosen for in-depth qualitative interviews based on their Jhpiego training, provision of maternal and newborn health services, and years of service. Provider interviews covered topics including antenatal and postnatal service utilization, integration of family planning and HIV services, and linkages to other levels of the health system.

Data quality was ensured by two field-based supervisors who provided overarching support to field implementation, including review of completed instruments and conduct of daily debriefings following in-depth interviews. Completed and supervisor-checked questionnaires were sent to Dar es Salaam for data entry and cleaning.

Qualitative provider interviews were digitally recorded, transcribed, and translated to English. Team debriefings at midpoint and endpoint of data collection reviewed emerging themes and assessed reliability of data through triangulation. After the midpoint debrief, revised interview guides focusing on emerging themes were implemented for the last seven health facilities visited by the research team.

### Analysis

This paper drew primarily from qualitative interviews with pregnant women and antenatal care providers on the topic of integrated HIV testing and counselling services during routine antenatal care. In addition, women’s and providers’ demographic profiles were also included as background information. Thematic qualitative data analysis was performed manually from a database coded and organized by Atlas.ti. Codes were derived from the structure of the interview guide and from themes that emerged during daily, midpoint and endpoint debriefings. Codebook development and coding were undertaken through consensus by a team, including research assistants who conducted data collection and whose work was reviewed by a supervisor. A framework approach [25] was taken in the qualitative portion of the research, utilizing an inductive approach with pre-defined research questions.

Preliminary findings from both the quantitative and qualitative analysis were shared with MoHSW and implementing partner for their feedback and review.

### Ethical approval

The study received ethical approval from the Muhimbili University of Health and Allied Sciences (MUHAS) and the Johns Hopkins School of Public Health (JHSPH) Institutional Review Boards. Permission to conduct the study was obtained from MoHSW and from the region and district administration authorities. Individual written consents were obtained from the study participants prior to their participation in the study. All information was kept confidential and anonymous.

## Results

### Profile of respondents and antenatal care seeking

We conducted a total of 203 clinical observations of pregnant women receiving routine antenatal care and 196 exit interviews with women. We received eight refusals for observations and seven refusals for exit interviews. In addition, we also conducted 65 quantitative interviews with providers of antenatal services and 57 qualitative in-depth provider interviews.

The mean age of pregnant women was 26.0 years, and their mean year of formal education was 5.8 years. The mean number of antenatal care visits that pregnant women had, including the one after which they were interviewed, was 2.7, and a majority of women came for antenatal care at the recommendation of their health provider. The mean time that women took to reach the health center was 48.3 minutes, while the mean time between arrival at the health center and being seen by the provider was 117.2 minutes (Table 4).

**Table 4:** **Characteristics of interviewed pregnant women (N=196)**

**Age, in years** (mean/median/range)	26.0/25.0/16 – 50
**Number of years of education** (mean/median/range)	5.8/7.0/0 – 13
**Previous number of live childbirths** (mean/median/range)	1.6/1.0/0 – 6
**Number of ANC visits completed** (mean/ median/ range)	2.7/3.0/1 – 9
**Number of weeks pregnant** (mean/median/range)	27.3/28.0/8 – 40
**Travel time between home and health facility, in minutes** (mean/median/range)	48.3/30.0/1 – 240
**Time between arrival at health facility and being seeing by provider, in minutes** (mean/ median/ range)	117.2/98.8/2 – 420
**Reason for attending ANC**	
Self-recommended	23.5%
Family member recommended	6.1%
Informal provider recommended	0.5%
Health provider recommended	52.0%
Complications this pregnancy	5.6%
Complications prior pregnancy	1.0%
Came to facility for other reason, then received ANC	1.5%
Other	27.0%

Some women expressed dissatisfaction with the long queues and wait times. For example, a 19-year-old woman during exit interview 07–08 said:

*[The] [n]umber of health providers should be increased so that we don’t need to spend a lot of time waiting for services.*

Researchers observed that although women at different health centers expressed this concern, most pregnant women saw the wait between their arrival and receipt of services as an expected part of care seeking at rural health centers.

Of the 65 health workers who were interviewed, almost 80% were female and the average age was 39.2 years. A little more than half were enrolled nurses, while registered nurses and medical attendants comprised 15.4% each. Antenatal care providers had worked for a mean duration 14.1 years in total, with a mean duration of 6.4 years at the facility where the interview took place (Table 5).

**Table 5:** **Characteristics of interviewed antenatal care providers (N=65)**

**Age, in years** (mean/median/range)	39.2/37.9/13 – 60
**Female**	78.5%
**Marital status**	
Married/Co-habitating	49.2%
Single	38.5%
Widowed/divorced/ separated	10.8%
**Designation**	
Assistant medical officer, 5 years of clinical training	1.5%
Clinical officer, 3 years of clinical training	6.2%
Assistant clinical officer, 3 years of clinical training	1.5%
Registered nurse, 4 years of nursing training	15.4%
Enrolled nurse, 3 years of nursing training	55.4%
Medical assistant, Secondary school	15.4%
Health assistant, Secondary school	3.1%
Other (“afisa muuguzi msaidizi,” assistant nursing officer)	1.5%
**Received in-service training**	
On HIV/AIDS	58.5%
On Focused ANC	31.3%
**Years as health worker** (mean/median/range)	14.1/11.0/0 – 39
**Years employed at this health center** (mean/ median/ range)	6.4/3.5/0 – 29
**Number of previous postings** (mean/median/range)	1.6/1.0/0 – 7

### Positive effects of integration on care seeking

According to both providers and pregnant women, provision of HIV testing during antenatal care has increased coverage to include more women and other populations that were not previously being tested, for example women from the Maasai community. In addition, integration has also increased the coverage of testing among men. Providers and women explained that they utilized the opportunity to test partners for HIV as well as to involve partners in counselling and MNCH in general. For example, enrolled nurse 02–28, a health worker since 1982, discussed the changes in the involvement of women’s partners in counselling as a result of PMTCT policy changes:

*…during the past a woman can come along with her husband but the man will stay outside*; *only if the woman has got some complications* [do] *we ask her to call her husband. Otherwise he will stay outside waiting for his wife until she finishes without gaining anything there… But nowadays if a woman comes with her husband, if there is education provided he will be involved too, due to the policy change even the man can enter and listen to the education provided.*

Providers and women noted that integrated services ensured that women were tested for and counseled about HIV during routine antenatal care visits, reducing the number of times they returned to the health center. Thus, integrated service delivery was more convenient for pregnant women. Provider 07–06, with 11 years of experience at the facility of interview, explained in this way:

[Providing HIV testing and counselling during ANC] *is a good service and very important to a mother because it saves time and a mother does not take a long time in getting the service. She is* [taking] *a pregnant test, then* [she will find out her] *HIV status, then she is getting back to her home place…*

Exit interview 02–07 with a 29-year-old woman who has had two previous live births confirmed this view:

[Providing HIV testing and counselling during ANC is] *good because if the provider tells me to come only for HIV instead of antenatal services I wouldn’t come.*

Pregnant women also appreciated knowledge gained during testing and counselling sessions about their health status and ways to prevent transmission from mother to child and between partners. Exit interview 13–11 with a 20-year-old woman who has had one previous live birth noted:

*I think* [HIV testing during ANC] *is a good system because you get a chance to know your HIV status so that if you’re infected, you can start treatment early. If you’re not, the counselling will help you take care of yourself.*

Moreover, provider 02–29, a health worker since 2008, commented that integrated HIV testing and counselling has reduced stigma, as the services became a routine part of the antenatal care expected during every pregnant woman’s visit to the health center. Provider 01–12, a health worker since 1989, added that integration of HIV testing and counselling into routine antenatal service delivery led to improved confidentiality for women. When a woman visited the health center for antenatal services, no one else knew her HIV status or her health issues. Provider 07–07 mentioned that having one health worker throughout the MNCH spectrum was reassuring to women and believed that women thought that having one provider ensured privacy of their information.

Aside from health advantages for mothers and children, integration has also given women an opportunity to exchange information with other women in the community. Provider 09–04, who has worked at the facility of interview for 23 years, explained the added benefit from peer learning:

*Another success* [of integration] *is* [that] *education has spread because when she gets knowledge here* [at the health center], *she convinces another mother also to test*; *that’s why you find many more mothers test than the fathers*, *because when you give knowledge here at the clinic about testing when she arrives*, *there she tells another mother; therefore we get many mothers.*

### Patient-provider interactions

Despite the perceived benefits of integrated HIV testing and counselling, care seeking-related challenges linked to the quality of provided services remained. One challenge that emerged surrounded patient-provider interactions, which included the nature of consent for opt out HIV testing, unequal social relations and lack of supportive communications between pregnant women and providers during counselling sessions, and privacy and confidentiality concerns.

According to providers, very few women refused HIV testing. In case of refusal, health workers continued to provide counselling for routine MNCH visits and for HIV testing during subsequent routine antenatal visits until women accepted. Pregnant women confirmed provider comments about the rarity of HIV test refusals and reported their understanding of consent for HIV tests in terms of provider authority rather than choice. Many women saw HIV testing as compulsory and did not know or were not counseled that they could opt out of HIV testing during antenatal services. Some women also reported that they felt that further antenatal care services would be withheld if they did not consent to be tested for HIV. In addition, some of the women also felt that they did not have an opportunity to voice their concerns and were disempowered from making informed decisions about consenting for HIV testing during antenatal services. For example, during exit interview 03–07, an 18-year-old woman with three previous live births expressed that she would have refused HIV testing had she known that it was an option available to her:

*…they did not tell me* [that I had a choice in HIV testing]*. If they did, I would say that I do not want to get an HIV test because I do not know the meaning* [of getting tested]*. I got tested, because I had no choice, and there is nothing you can do about it. You just follow the instructions of the providers.*

Provider 15–04, with 13 years of experience at the facility of interview, acknowledged that refusal was difficult for women to voice, since women interpreted the HIV test as “orders from the Ministry of Health” and “she cannot violate an order without a reasonable cause.” Women’s responses also showed that providers were highly regarded, and that providers’ positions of authority gave them power to make health-related decisions for the women. Many women viewed HIV testing as following the instructions of the providers, which, to the women, were beyond questioning.

This perception of the provider as the person to make health decisions for the women was seen in other aspects of the antenatal care consultation. From exit interviews, 52.0% of pregnant women reported that they came for antenatal care at the recommendation of their providers, in comparison to 23.5% who responded that they chose to come of their own volition (Table 4). A 25-year-old woman with two previous live births in exit interview 14–06 expressed her thoughts on being asked by the provider to return to the health center again and again due to an HIV test kit stock out: “We just think it’s okay. What can we do then? We can do nothing. We just go.”

Only a few pregnant women discussed health decisions in terms of exerting control over their own bodies. The few women who did so articulated an intentional involvement in decision-making and understood health services as beneficial to their health. For example, when probed about whether she felt comfortable telling the provider her opinion about HIV testing, a 28-year-old woman with three previous live births responded in exit interview 13–05:

*Yes, that is my body if it is going to be tested. So it does not benefit* [the provider] *if she knows my health; it benefits me.*

Most of the women who expressed this perspective came from the few facilities where women reported supportive relationships with their providers and felt encouraged to ask questions.

In contrast, many women at other facilities communicated discomfort in voicing their opinions or asking questions of their providers due to the providers’ “harsh” attitude. Patient-provider relations were complicated by differential social status, including dramatic divergence in educational levels and an average age difference of 14 years. Findings from the observations of antenatal consultations showed that while at least 89.7% of providers greeted women and her companions with respect, spoke using understandable local language, and addressed women respectfully, only 66.5% of women were encouraged to ask questions and only 20.8% of providers responded to their questions (Table 6). In addition, only 28.6% of providers thanked the women for coming to the health center for services.

**Table 6:** **Observations of interactions between women and providers during antenatal services (N=203)**

Provider greets woman and her companion/relative with respect	89.7%
Provider speaks using easy, understandable local language	99.0%
Provider addresses the woman by her name/calls her ‘mama’	93.1%
Women encouraged to ask questions during clinical session	66.5%
Provider respond to questions asked by women	20.8%
Provider thanks woman for coming to health facility for services	28.6%

At the same time, some health workers were friends with women in the community, which enabled a pregnant mother to seek services at the facility. Provider 06–06, a health worker since 1980, said:

*women* [seeking services at the health center] *can be my friends and I have their phone number… When they reach here,* [they say]*… ‘*[W]*here is the* [specific] *attendant, I am looking for attendant’… You already know this medicine is a private thing…so you come, you give her.*

While this relationship could be positive, some women at one facility also reported that they could not trust their providers to keep their HIV results confidential and expressed concern that the provider would discuss their results with other providers at the facility and with community members at home. These concerns did not vary by educational levels. At this particular facility, nine of the ten interviewed women completed seven years of formal education, or primary schooling, and one woman completed 11 years of formal education. Provider 07–06, a health worker since 1998, was aware that women did not trust their providers to maintain confidentiality in some cases. During antenatal services at one facility, a research team member observed providers openly discussing a pregnant woman’s HIV+ status and medication needs in public.

### Stigma

Another challenge that emerged from interviews with women and providers was stigma surrounding HIV infection at both the individual and community levels, resulting in some women seeking health services in other health facilities or through other providers, for example community health workers or traditional healers.

Despite integration of HIV testing and counselling in routine antenatal visits and the reduction of stigma associated with HIV testing, fear of a positive test result was still a significant barrier to care seeking. A 21-year-old woman with one previous live birth in exit interview 06–05 commented:

*Some* [pregnant women] *are afraid to know their results, as they know that being HIV positive is the end of living.*

Women’s fear of a possible positive result was strong enough that provider 02–28 and three women described it as a contributing factor to pregnant women deciding against attending antenatal services. A 22-year-old woman in exit interview 07–03 commented that women who opted out of antenatal services at the facility would rather not know their HIV status than to subject themselves to the psychological pressure of thinking that they would die soon after a positive test result, even though treatment was generally known to be available. A 21-year-old woman with no previous live births in exit interview 08–02 noted:

*Other* [women] *are afraid to take their results from the HIV test while others do not come back once they are tested and found to be HIV positive…they are afraid of people hearing about their HIV status. In the community, those who are HIV positive are ostracized. So they are afraid to go back and forth to the health center.*

Some women reported knowing other pregnant women who had undergone HIV testing during their first antenatal visit and then discontinued further attendance at the facility. Providers added that some women would go to other points of access, such as dispensaries and community health workers, for antenatal services.

## Discussion

This study found that both pregnant women and providers had positive perceptions of the integration of HIV counselling and testing into antenatal services. Many providers and pregnant women felt that integration has increased uptake of HIV testing and enabled marginalized groups and partners to be involved. In addition, some women stated that knowing one’s status would help protect both the pregnant woman as well as her unborn child, and that early testing and treatment were key to preventing mother-to-child transmission. Yet potential gains in increased coverage of HIV testing belied outstanding challenges related to perceptions of patient-provider relations and stigma against HIV. The implicitly compulsory nature of the HIV test described by respondents raised questions about consent. A sense of powerlessness and anxiety was evident in women’s discussions about providers’ behavior. Women were concerned that providers were unable to maintain confidentiality of their HIV status at the health center and in the wider community. Stigma and fear of implications of a positive test result discouraged some pregnant women from seeking antenatal services completely or contributed to their discontinuing antenatal services at the health center after HIV testing.

This analysis had some limitations. The cross-sectional design of the study and timing of data collection during the harvest season in an agricultural region could potentially exclude pregnant women with harvesting responsibilities who did not attend antenatal clinic during the time period when the data collection team was at each health center. Furthermore, informant fatigue could have resulted from long in-depth interviews, which usually followed quantitative interviews. This fatigue was mitigated by rest periods between the quantitative and qualitative portions of interviews, ranging from half an hour to an entire day.

As ANC is the entry point for PMTCT services, engaging with women from their first antenatal care visit is important. From this study, women reported that HIV testing during antenatal services was generally accepted by pregnant women, confirming other studies that found generally positive attitudes towards integrated HIV testing and counselling [26,27]. In South Africa, women who knew their HIV status were more likely to utilize health services and adhere to drug regimens across the MNCH spectrum [28], suggesting that knowing HIV status from integrated HIV testing and counselling could lead to better drug adherence if treatment were required. In our study, we also found that with integrated service delivery, women’s exchange of information in their communities included HIV counselling, which potentially reinforced HIV counselling messages discussed during antenatal visits and community outreach efforts and furthered women’s knowledge about HIV.

At the same time, most of the women in this study believed that HIV testing was compulsory and were not aware of the “opt out” option, with some perceiving HIV test as necessary before receipt of antenatal services. This finding was also reflected by other studies in the region [29,30]. In fact, some women found HIV testing to be coercive and that they were unable to make an informed decision [31,32]. In rural Malawi, an HIV-positive result from testing during routine antenatal care discouraged some women from coming back for further services and from delivering at the health center [30]. In this study, the disparity in providers’ and pregnant women’s views about consenting for HIV testing holds implications for service utilization further along the PMTCT cascade and MNCH continuum of care by HIV-positive women.

The perceived compulsory nature of HIV testing was complicated by the relationship between providers and women seen in this study, which was often characterized by unequal social relations and lack of supportive communication. This study found that women often accepted the recommendations of providers without question, which emerged from their understanding that providers were best equipped to make health decisions on their behalf because providers knew more about health issues. Additionally, while some women and providers reported that they shared positive and supportive relationships at the health center and in the community, other women perceived their providers to be harsh, rude, and sometimes dismissive. Studies from Burkina Faso, Kenya, Malawi, and Uganda had similar findings and reported that women perceived health workers to be powerful figures who made decisions regarding their health, and that this message was reinforced by social, cultural, and political norms [30,32].

In addition to some participants highlighting poor consent processes and lack of supportive communications, some women in this study were concerned about their providers breaching confidentiality by discussing their HIV status with other providers or with community members. Other studies in the region echoed this finding [32,33]. While in general, providers kept the confidence of women seeking health services [27], direct breaches of confidentiality by providers have been documented in the literature [30,32,34]. In one study in Tanzania, a health worker demanded a bribe from a woman in return for keeping her information confidential [33]. A trusting and informative provider-patient relationship has been shown to be a key factor not only in the perceived quality of services but also in the uptake of further PMTCT services, drug adherence, and health outcomes [28,31,35]. Aside from ethical concerns, direct breaches of confidentiality by providers has also contributed to women’s distrust not only of the providers but also of the health system [33]. At the same time, another study aiming to measure differences in consent, confidentiality, and referral between non-integrated voluntary counselling and testing programs and integrated PMTCT programs found ethical practices of testing and counselling in general and no difference in confidentiality practices by the mode of HIV testing and counselling as perceived by women [27]. The literature thus points to mixed observations regarding confidentiality and suggests that patient-provider relations may be problematic independent of integration.

This study found that the reported increased uptake of HIV testing could be partly attributed to de-stigmatization of the test, as it became a routine part of and accepted by most women attending antenatal services [36]. Improved access to and coverage of HIV testing could be seen in the increasing percentage of women and men who were tested for HIV in the past 12 months and received their results: 19% to 30% for women and 19% to 27% for men from 2007–2008 to 2011–2012 [2]. However, multiple studies found that stigma against people living with HIV and AIDS was still prevalent, and that anxiety and fear surrounding a potentially positive HIV test result continued to deter women from seeking care [37]. In a study conducted in Dar es Salaam exploring the feasibility of Option B+ as recommended by the WHO, stigma remained an important barrier to care and treatment as well as adherence to the drug regimen for HIV+ women [38]. In rural communities, stigma has an even stronger influence over women precisely because women in rural areas, in comparison to women in urban areas, have fewer choices in access points for health services. Thus, since stigma has remained a barrier for uptake of the PMTCT service cascade [39,40], de-stigmatization serves as an important first step to address “missed opportunities” [41–43] in engaging with women seeking MNCH services in order to prevent vertical transmission.

Evidence shows that community engagement is necessary for reducing stigma and promoting a supportive social environment in which women and their families can seek care [34,44]. In Rwanda and Kenya, early engagement with community stakeholders has contributed to the success of similar integrated and community-based PMTCT programs [44,45]. In the Democratic Republic of Congo, community leaders led efforts to identify HIV- and PMTCT-related priorities, and collaboration between community health workers and facility providers ensured that pregnant women could access the services they needed across a continuum of care [46]. The success of scaled up PMTCT programs in these countries highlighted the importance of collaboration between the community and the health center in reducing stigma surrounding HIV/AIDS and creating a continuum of care for all pregnant women.

At the health center, a supportive relationship between providers and women must be fostered to address concerns about the nature of HIV testing and confidentiality. Revising the content of in-service training, facilitating follow up provider training, and conducting community outreach with emphasis on a patient rights-based approach would be first steps in enabling a more equal and trusting relationship between providers and women. This relationship has been recognized as key in promoting women’s health [47]. In the Health Workers for Change program, for example, the participatory quality improvement measure improved relations between providers and patients [48]. In addition, the Skilled Care Initiative in Burkina Faso, Kenya, and Tanzania showed that “compassionate care” could contribute more broadly to quality of care and utilization of services over the entire MNCH spectrum [49]. However, while quality improvement initiatives could be effective, the determinants of success were not always clear [50–52], and the initiatives were not always successfully scaled up by Ministries of Health. Change can only be maintained if a well-equipped health system is responsive to needs of women and providers in health centers. From this perspective, a health systems approach to addressing this challenge requires a new consideration of “dignified, respectful health care” [53] as countries strengthen the integration between PMTCT and MNCH programs.

## Conclusion

This study found that while perceptions of integrated service delivery of HIV services and antenatal care were generally positive, important barriers related to patient-provider interactions and stigma remained for women seeking antenatal care at health facilities. Tanzania and other low-resource, high-burden countries are currently rolling out Option B+, which further integrates care and treatment into maternal health services. In this context, women’s care seeking practices and perceptions about integrated health services are especially important to understand so as to inform future policies and service delivery reforms. The identified challenges must also be mitigated to ensure that pregnant mothers actively engage with the MNCH spectrum of care for themselves and their children, and concerns about integrated delivery of care must be addressed in order to reduce the impact of HIV/AIDS and to improve maternal and child health in Tanzania.

## Endnote

^a^At the time of data collection, Gairo had yet to become an independent district and facilities in the district were counted as part of Kilosa district.

## Abbreviations

ANC: Antenatal care
ART: Antiretroviral therapy
CTC: Care and treatment center
FANC: Focused antenatal care
HIV/AIDS: Human immunodeficiency virus/acquired immune deficiency syndrome
JHSPH: Johns Hopkins School of Public Health
MCH: Maternal and child health
MNCH: Maternal, newborn and child health
MoHSW: Ministry of Health and Social Welfare
MUHAS: Muhimbili University of Health and Allied Sciences
PMTCT: Prevention of mother-to-child transmission
RCH: Reproductive and child health
WHO: World Health Organization
